# Penile gangrene: an unusual complication of malignant priapism in a patient with renal cell carcinoma

**DOI:** 10.11604/pamj.2019.34.130.20447

**Published:** 2019-11-05

**Authors:** Mohammed Aynaou, Amine Elhoumaidi, Tarik Mhanna, Paapa Dua Boateng, Mehdi Chennoufi, Ali Barki

**Affiliations:** 1 Department of Urology, Mohamed VI University Hospital Center, Mohamed First University, Oujda, Morocco

**Keywords:** Penile gangrene, malignant priapism, renal cell carcinoma

## Abstract

A 68-year-old man presented with priapism and penile gangrene. The patient had no history of penis trauma or medications for erectile dysfunction. Corpus cavernosa aspiration cytology were positive for malignant cells. Total penectomy was performed. Enhanced chest and abdominal computed tomography showed a left renal tumor with pulmonary and hepatic metastases. Ultrasound-guided renal biopsy showed clear cell renal cell carcinoma.

## Introduction

Penile gangrene is an infrequently encountered clinical entity and an unusual complication of priapism [1-4]. There are case reports of penile gangrene resulting from various etiological factors like diabetes mellitus, chronic renal failure, penile strangulation etc. [5, 6]. Penile metastasis mimicking priapism is extremely rare [7]. We present a case of cell renal carcinoma revealed by penile gangrene complicating priapism.

## Patient and observation

**Figure 1:**
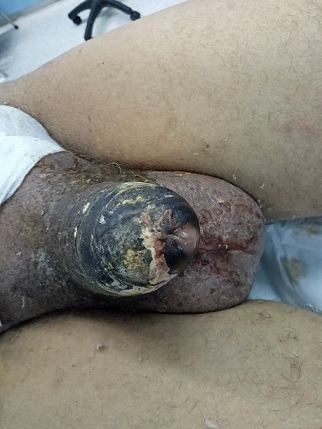
Total gangrene of the penis

**Figure 2:**
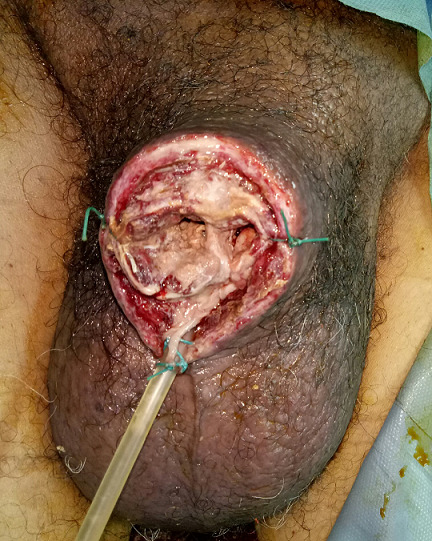
Post-operative picture of total penectomy

**Figure 3:**
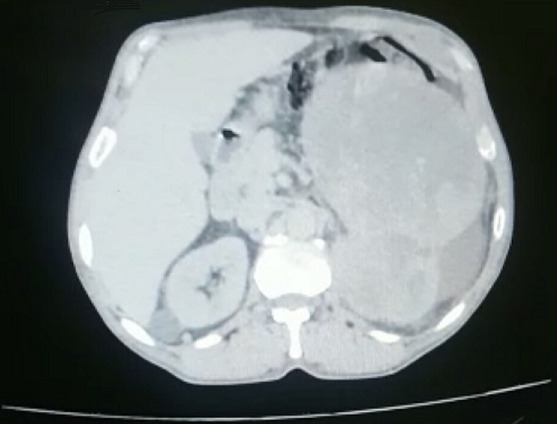
CT scan showing left renal tumor

A 68-year-old presented with a four weeks history of painful, persistent erection and inability to pass urine. He denied any preceding intake of erection-enhancing medications or exposure to trauma. He was not a known sickle cell disease patient. He admitted to a history of weight loss, asthenia and loss of appetite. However, he presented with no hematuria or abdominal pain and had never been admitted in the past. Examination revealed that the penis was erect to about 90°, rigid, stained and necrotic (Figure 1). Hemoglobin was 10 g/dl, the white cell count was 7. Electrolyte, urea and creatinine were normal. Total penectomy was performed (Figure 2). Corpus cavernosa aspiration cytologie were positive for malignant cells. Enhanced chest and abdominal computed tomography (CT) showed a left renal tumor with pulmonary and hepatic metastases (Figure 3). Renal mass biopsy revealed clear cell renal cell carcinoma. The patient was transferred to the oncology department. Unfortunately, he continued to deteriorate and died of his disease 4 months later.

## Discussion

Malignant priapism is a term first use by Peacock to in 1938 to describe persistent, non sexual erection caused by invasion of malignant cells into the cavernosal sinuses and their associated venous systems [8]. Penile metastasis is extremely rare. More than 69% of metastases are from bladder, prostate and rectosigmoid cancers. There are followed by kidney cancer with a ratio of 6.9% [9]. Various mechanisms for penile metastasis have been suggested, which include arterial spread, retrograde venous, lymphatic route, direct extension and possibly implantation of instrumentation [10]. The diagnosis of penile metastasis can be confirmed using several modalities that include CT, magnetic resonance imaging (MRI), cavernosography and biopsy of the corpus cavernosum. Penile MRI is an excellent modality for the detection of hemorrhage and thrombosis, and for imaging the cavernosal vessels [11].

## Conclusion

Penile metastasis carries a poor prognosis and treatment is usually palliative. Partial, total penectomy or even radiotherapy may be required.

## Competing interests

The authors declare no competing interests.
